# Reliability Testing Procedure for MEMS IMUs Applied to Vibrating Environments

**DOI:** 10.3390/s100100456

**Published:** 2010-01-07

**Authors:** Giorgio De Pasquale, Aurelio Somà

**Affiliations:** Department of Mechanics, Politecnico di Torino, Corso Duca degli Abruzzi 24, 10129 Torino, Italy; E-Mail: aurelio.soma@polito.it

**Keywords:** IMU, MEMS, navigation systems, reliability, accelerometers, aerospace

## Abstract

The diffusion of micro electro-mechanical systems (MEMS) technology applied to navigation systems is rapidly increasing, but currently, there is a lack of knowledge about the reliability of this typology of devices, representing a serious limitation to their use in aerospace vehicles and other fields with medium and high requirements. In this paper, a reliability testing procedure for inertial sensors and inertial measurement units (IMU) based on MEMS for applications in vibrating environments is presented. The sensing performances were evaluated in terms of signal accuracy, systematic errors, and accidental errors; the actual working conditions were simulated by means of an accelerated dynamic excitation. A commercial MEMS-based IMU was analyzed to validate the proposed procedure. The main weaknesses of the system have been localized by providing important information about the relationship between the reliability levels of the system and individual components.

## 1. Introduction

Monitoring of dynamic parameters in systems and vehicles is a basic issue for two main reasons: (1) instantaneous acceleration, velocity, attitude, *etc.*, must be measured to provide feedback signals for the stability controllers and (2) vibration levels must be checked to prevent the failure of components and make a diagnosis on their residual lifetime. Inertial sensors can be suitably employed for both the purposes as single sensors or can be assembled in inertial measurement units (IMU). The conventional piezoelectric or capacitive accelerometers are largely used for the measurement of vibrations, but the emerging technology of micro electro-mechanical systems (MEMS) can be a valid alternative owing to some advantages related to their small size. Many attractive peculiarities can be observed from the application of microsystems technology to sensors, for example, the small supplied power needed, the small weight, and above all, the low cost of each unit. The MEMS accelerometer costs about 10% of the cost of one of the cheaper traditional accelerometers available in the market; the average price across the different applications was $2.50 in 2004 and is expected to be less than $1.90 in 2010, following a trend of price erosion [1]. A number of works have been proposed regarding MEMS accelerometers [2–8] and gyroscopes [9,10], particularly focusing on their fabrication processes, packaging, dynamic characterization, calibration, and effects of environment on their functioning. In addition, the integration of different MEMS sensors in more complex IMU for avionics has also been discussed [11–16], relative to their applications in satellites, helicopters, unmanned air vehicles (UAV), micro air vehicles (MAV), *etc.* The development of IMU based on MEMS technology for applications in the aerospace field as attitude controllers is currently under investigation, with the integration of accelerometers, gyroscopes, inclinometers, altimeters, and GPS navigators [17–19]. However, not enough information is available to define the reliability of MEMS inertial sensors and IMU, thus, representing a serious lack of knowledge considering the specific application for which they are designated. In fact, the on-board installation of any sensor is subordinated to carry out severe tests defined by standard normative. The safety levels typical for aerospace field require the development of inertial navigation sensors capable of maintaining their performances throughout the time of flight in terms of stability and accuracy, even in the presence of severe environmental vibrations. The application of inertial sensors in UAV or MAV environment are characterized by several challenging navigation problems, as highlighted in several studies [18,19]. First, the navigation system must operate in a working environment dominated by high levels of vibrations, exposed to atmospheric elements and severe temperature gradients. An additional difficulty is that it is usually constrained in size, weight, and cost. Sometimes, the magnetic interferences affect the compass, and the GPS may lose its functionality temporarily, but inertial navigation must remain functional. Under these considerations, the fundamental question no longer relates to the original performances of the sensor, but rather, is the MEMS-based IMU capable of preserving its original performances during its in-field functioning?

Some works have discussed the reliability of IMUs [20–23]. The works reported in the literature state that the commercial MEMS sensors generally have good reliability, due to both good electro-mechanical properties of polysilicon and protective polymeric packaging. Dedicated experiments have been conducted on single sensors using small test benches for calibration, characterization, and reliability of evaluation; for this last case, all the results testified that the electronic circuit used for the sensor supply and control fails before the sensor itself [24]. However, the evidence that the reliability level of commercial MEMS sensors is high does not assure that the same property is valid for an IMU. The coexistence of many sensors per IMU increases the global chance of failure of the whole unit; the presence of electrical interfaces and connections, and additional electronic devices generally determine a decay of reliability. Following a bottom-up approach, the reliability of MEMS can be considered on three different levels: material reliability, component reliability, and system reliability. For navigation systems, these levels correspond to the reliability of polysilicon, constituting the movable sensing microstructures (material) of each inertial sensor, such as accelerometers or gyros (component), and the IMU (system). Some studies presented by the authors on the material’s level of reliability are already known in the literature, and are focused on the behavior of gold under mechanical fatigue in microscale [25,26]. In the present study, MEMS inertial sensors’ reliability related to component and system levels is investigated.

This work proposes a procedure to test the reliability of an IMU based on MEMS for applications in aerospace and vibrating environments. Standard procedures for sensors’ calibration and signal analysis were used and combined with a dynamic excitation test to reproduce the actual working conditions. A commercial IMU for aerospace applications was tested according to the procedure presented; the reliability analysis provided by the procedure confirmed the effective behavior of the IMU and its components. The main problems affecting the sensor were identified and some conclusions about the sensing errors and system weaknesses were in agreement with some in-field experiences reported in the literature [27–29].

## 2. Analysis of Signal Components

Position, velocity, acceleration, and attitude of aircrafts are usually monitored by an inertial navigation system (INS) that bases its functioning on the processing of signals provided by an IMU. The definition of sensor characteristics in terms of operating range, scale factor accuracy and linearity, bias, axis alignment stability, and output noise is fundamental to fit the aerospace requirements; some reference values for the sensing parameters required are reported in [30]. The range of sensing performances required by three different applications in the aerospace field with increasing accuracy specifications are indicated in Table 1. The reported values were obtained from a survey among commercial devices.

The main error sources affecting the measure of an IMU are: (a) inertial sensor imperfections, (b) incorrect navigation system initialization, and (c) imperfections in the error model. The first type of error is caused by bias, scale-factor instability and non-orthogonality of axes, the second type is caused by alignment inaccuracies, and the third type is caused by the approximations affecting the algorithm used in the analytic model of the signal [31,32]. The acceleration (*l_a_*) measured by the IMU on each axis can be described by the following equation:

(1) $$l_{a}=a+b_{a}+S_{a}a+\mathrm{Ta}+\gamma +d\gamma +\varepsilon_{a}$$

where *a* is the true acceleration, *b_a_* is the sensor bias, *S_a_* is the scale factor (or acceleration gain), *T* is the non-orthogonality factor between the axes (cross-coupling), *γ*+*dγ* are the average acceleration of gravity and its variation, respectively, and *ε_a_* is the sensor noise. A similar equation can be used to describe the angular velocity (*l_ω_*) measured by the IMU on the single axis:

(2) $$l_{\omega }=\omega +b_{\omega }+S_{\omega }\omega +T\omega +\omega_{e}+\varepsilon_{\omega }$$

where *ω* is the true angular velocity, *b_ω_* is the sensor bias, *S_ω_* is the scale factor (or angular velocity gain), *ω_e_* is the Earth angular velocity, and *ε_ω_* is the sensor noise. The values of coefficients *b_a_*, *b_ω_*, *S_a_*, *S_ω_*, and *T* are usually not constant throughout the process, but are subjected to small variations around their average level. The nominal value of these parameters can be determined by means of a sensor calibration (e.g., *six positions static test*) before it is being used, and fixed as a default value. The variation of their actual level with respect to the default value can be monitored by additional in-run calibrations during the functioning.

Depending on the variation of the calibration parameters (especially, bias, scale factor, and cross-coupling), the measures may be affected by a systematic component of error. Subsequently, an accidental component of error may be present owing to the sensor noise. Both the reported sources of error are superimposed on the measured signal [33]. The *bias* is a systematic error present in all the measurements; it represents the main source of error and can be divided into a static and dynamic factor. The first component is called a *fixed bias*; it includes the *run-to-run* variation and any residual bias after the IMU calibration, and takes into account the fact that these errors are not really constant, but may fluctuate each time the instrument is switched on. The dynamic component, also known as the *in-run bias*, represents the variability in short time periods (around 1 min long) and is related principally to the temperature-change sensitivity of the sensor. The *scale factor* is a measure of the relationship existing between the output of the sensor and the true value of the physical quantity measured. Within this error, the contributions of the components to lower orders of magnitude, such as the *scale-factor non-linearity* and the *scale-factor asymmetry* exist. The *cross-coupling* error is related to the misalignment of the “physical” axes of the inertial sensor with respect to the theoretical reference system [32]. This inaccuracy is mainly owing to assembling tolerances, and makes each accelerometer/gyro partially sensitive to the forces acting along the two orthogonal axes. Furthermore, the non-orthogonality of the axes also results in an additional scale factor, which is typically two to three orders of magnitude lower than the actual error of non-orthogonality. All the inertial sensors are also more or less sensitive to a number of accidental errors, which may originate externally (interference) or internally (noise) to the measurement system.

## 3. Reliability Test Description

The procedure described is suitable for monitoring the sensing performances of an IMU during its operative functioning in vibrating environments. The procedure is based on the simulation of real vibrating conditions by means of a dedicated spectrum of excitation. The variation in systematic and accidental errors caused by the dynamic excitation is calculated using standard approaches included in the proposed procedure. The reliability test for inertial navigation systems and IMU based on MEMS sensors comprises the following three steps:

1—first static calibration and Allan variance (AV) calculation
2—dynamic excitation
3—second static calibration and AV calculation

### 3.1. Static Calibration

The static calibration allows estimating the systematic component of errors affecting the signal. This calibration must be performed both before and after the dynamic excitation (Step 2) to detect error variations owing to eventual sensor damage caused by vibrations. The *six positions static test* can be usefully applied to calibrate the IMU. It requires orientating each axis of the sensor, both upwards and downwards, in the vertical position, having a total of six configurations. The signal is acquired at each position of the sensor for 20 min.

The sensor bias (*b_a_*) can be calculated using the sum of the accelerations detected by the sensor in the opposite directions of the same axis; if a horizontal axis (γ = 0) is used, then the bias can be obtained as:

(3) $$\frac{{\left[l_{a}\right]}_{x}+{\left[l_{a}\right]}_{-x}}{2}=\frac{{\left[b_{a}\right]}_{x}+{\left[b_{a}\right]}_{-x}}{2}=\frac{{2b}_{a}}{2}=b_{a}$$

where [*l_a_*]_x_ and [*l_a_*]_−x_ are the accelerations measured in the *x* and −*x* directions, respectively. The scale factor can be computed on the vertical axis (where γ ≠ 0) as:

(4) $$\frac{{\left[l_{a}\right]}_{z}-{\left[l_{a}\right]}_{-z}-2\gamma }{2\gamma }=\frac{\left(\gamma +{\left[b_{a}\right]}_{z}+{\left[S_{a}\right]}_{z}\gamma \right)-\left(-\gamma +{\left[b_{a}\right]}_{-z}-{\left[S_{a}\right]}_{-z}\gamma \right)-2\gamma }{2\gamma }=\frac{{\left[S_{a}\right]}_{z}\gamma +{\left[S_{a}\right]}_{-z}\gamma }{2\gamma }=\frac{{2S}_{a}\gamma }{2\gamma }=S_{a}$$

where [*l_a_*]_z_ and [*l_a_*]_−z_ are the accelerations measured in the *z* and −*z* directions, respectively. Reported relations assume that both bias and scale factor are equal in the opposite directions of the same axis (*i.e.*, [*b_a_*]_x_ = [*b_a_*]_−x_ and [*S_a_*]_x_ = [*S_a_*]_−x_). Thus, it is possible to define the bias and the scale factor for each axis by rotating the sensor.

### 3.2. Allan Variance

The AV is a simple and efficient method to identify and characterize different stochastic processes and their coefficients, allowing estimation of the accidental component of errors that affect the signal [34,35]. Through some simple operations on the sensor outputs, a characteristic curve of the AV can be obtained and further used to determine the types and magnitudes of errors affecting the data [36,37]. If *N* is the number of samples from an inertial sensor with a sample time *τ_0_*, then a group of *n* data points can be created (with *n* < *N*/2); each group member is called a cluster τ with size *nτ_0_*. If the instantaneous output of the sensor is assumed as *Ω* (*t*), then its corresponding integration (e.g., for the gyro output) is the angle:

(5) $$\theta \left(t\right)=\int_{t}\Omega \left(t\right)\mathrm{dt}$$

The angle of the sensor is measured at discrete times given by *t* = *k*τ_0_ (for *k* = 1, 2, 3,..., *N*). By using the notation *θ* (*t*) = *θ* (*k*τ_0_) = *θ_k_*, the average angle between the times *k*τ_0_ and (*k*τ_0_ + τ) is given by:

(6) $${\bar{\theta }}_{k}\left(\tau \right)=\frac{1}{\tau }\int_{{k\tau }_{0}}^{{k\tau }_{0}+\tau }\Omega \left(t\right)\mathrm{dt},\;\;\;\;\;\tau =n\tau_{0}$$

The AV, estimated from a finite number of samples, is defined as follows:

(7) $$\sigma^{2}\left(\tau \right)=\frac{1}{{2\tau }^{2}\left(N-2n\right)}\sum_{n=1}^{N-2n}{\left(\theta_{k+2n}-{2\theta }_{k+n}+\theta_{k}\right)}^{2}$$

All the definitions for the gyroscopes given earlier are also valid for accelerometers. There is a very important relation between the AV and the power spectral density (PSD) of the random processes, given by the equation:

(8) $$\sigma^{2}\left(\tau \right)=4\int_{0}^{\infty }S_{\Omega }\;\left(f\right)\frac{{\text{sin}}^{4}\;\left(\pi f\;\tau \right)}{{\left(\pi f\;\tau \right)}^{2}}\mathrm{df}$$

where S_Ω_ (*f*) is the PSD of the process *Ω* (*t*), representing the output signal of the sensor in this case. In the derivation from equation (8), it is also assumed that the random process *Ω* (*t*) is stationary.

**Figure 1.**
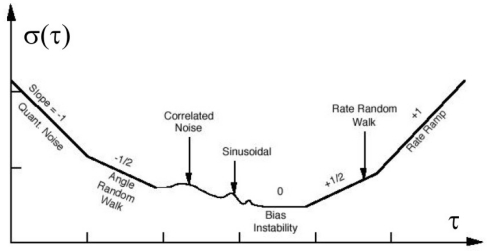
Generic Allan deviation plot in the clusters domain [36].

The most attractive feature of AV is the ability to define various error components by the slope of the root AV (*i.e.*, the Allan deviation) plot in the clusters domain; typical errors affecting inertial sensors, which are detectable through the AV, are the quantization noise, angle random walk, correlated noise, sinusoidal noise, bias instability, rate random walk, and rate ramp (Figure 1). Correlated and sinusoidal noises have minor contributions to the total noise, and they appear only at long-time clusters; all the other errors are believed to have the most impact on the MEMS sensors [38].

### 3.3. Dynamic Excitation

Highly accelerated life testing (HALT) and highly accelerated stress screening (HASS) are well-known procedures able to verify the operational limits of a system or the weaknesses of its components. During these tests, the temperature and vibration limits of the system are exceeded until either a failure occurs or the limits of the testing chamber are reached. The goal of these procedures is to find the weakest links inside the system, so that they may be improved or eliminated. In the first part of the procedure (*step testing*), only one environmental variable is tested to the extreme, *i.e.*, either temperature or vibration; once there is a failure, the cause of it is determined and the unit is fixed to continue the test. The second part of the procedure (*combination testing*) combines temperature and vibration that are both varied; again, modifications are made and testing is continued until it is either no longer economically or physically feasible or the limits of the test apparatus are reached. Failures are analyzed and new corrective actions on the electronic circuit are performed. The failure may have two different sources: *operational* (if the processing of sensed data is affected, typically related to the loss of calibration or to malfunctions at software level) or *destructive* (if the sensors or electrical components are compromised). Both types of failure imply the loss of functioning of the IMU; the second one is related to connectivity issues, broken leads, intermittent contacts, component malfunctions, and solder issues [39] and necessitate the recovery of hardware components to restore the functioning.

A dynamic excitation has been used in this study to simulate the environmental vibration acting on the sensor. The imposed vibration spectrum must reproduce the actual working conditions as faithfully as possible. In general, the effective amount of energy transferred from the outside to the sensor is rather difficult to determine in the case of random vibrations. A possible strategy consists of measuring the vibration spectrum of the working environment and reproducing it by means of an electro-mechanical testing system. However, this approach requires a direct access to the final machine or system on which the sensor will be applied, which is not always available. Thus, a faithful reproduction of the real vibration spectrum requires a very long time of testing, which is not suitable for sensor validation in realistic conditions of work. The use of standard procedures to simulate vibration effects on the IMU is advantageous owing to the following reasons: (a) their standardization allows a simpler implementation and an easy sharing of the results; (b) these procedures can often be identified as “accelerated tests” and are consequently able to reduce the testing time; (c) they are defined to reproduce the most severe conditions for a specific application providing a conservative result; (d) they are defined for several working environments depending on the final application (aerospace, automotive, robotics, buildings, human, *etc.*); and (e) they allow the standardization of the global validation process for the IMU.

**Figure 2.**
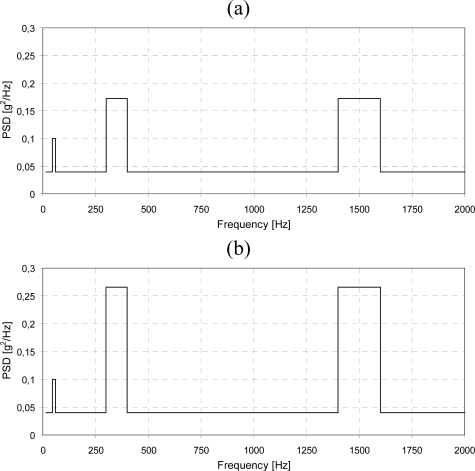
Level of vibration spectra for (a) functional and (b) endurance sections. The narrow band boundaries are 45–60 Hz, 300–400 Hz and 1,400–1,600 Hz in both cases.

The vibration spectrum excitation applied has been derived from MIL-STD-810-E normative [40]. It is composed of three separate parts: the *functional section* that is repeated at the beginning and end of the procedure, and the *endurance section*. The levels of vibration spectrum for functional and endurance sections are reported in Figure 2a,b, respectively. In both cases, they are characterized by a broadband sinus vibration ranging from 15 to 2,000 Hz, and three superimposed narrow bands of higher amplitude level; the maximum level of vibration is reached during the endurance section. The whole dynamic excitation must be repeated along the three axes of the IMU. Each functional section is 30-min long, while the endurance section is 5 h long; the sweep rate and the level of excitation for both the cases are reported in Table 2 for the broadband vibration as well as for each narrow band. The whole test is completed in 18 h, if the three axes are considered. The complete test plan is reported in Table 3.

## 4. Procedure Validation

### 4.1. Description of the IMU

**Figure 3.**
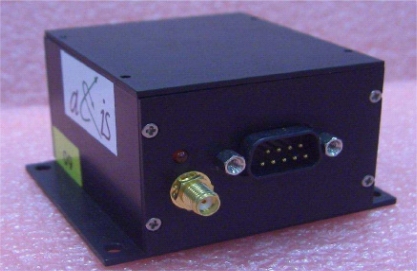
The AXIS-AIS402 inertial measurement unit.

The reliability test described in the previous section was validated on the IMU AXIS-AIS402 [41] for applications in aeronautics (UAV and MAV), robotics, and automotive navigation, shown in Figure 3. The IMU was fabricated using low-cost MEMS sensors with medium-high performances. It was able to provide static and dynamic information about the fundamental flight parameters, such as positioning (latitude and longitude), altitude, attitude, heading, acceleration, and angular velocity along the three axes. The sensing unit comprised two series of accelerometers (2 g and 15 g full-scale), three rate gyros, a 3-axes magnetometer, and a GPS receiver on 12 channels. The main characteristics of the IMU are reported in Table 4. The procedure for reliability testing was validated using the acceleration outputs of the IMU.

The output signals are addressed to a PC in the form of separate packets of data, so that the number and type of sensing channels can be set before the acquisition. Furthermore, the frequency at which data are transmitted from the IMU to the host can also be changed according to the specific application, up to a maximum value of 100 Hz. The setting configuration can be imposed by the user by means of a software interface, and can subsequently be transferred to the IMU through the dedicated communication protocol; the measured data are stored in a binary file during acquisition, and successively converted into ASCII format. A user-friendly output interface is created using LabView8.5 reporting the aircraft attitude and other flight parameters. Some information about the sensing performances of the IMU is reported in Table 5.

### 4.2. Experimental Setting

The six-position static test was performed to calculate the systematic components of errors. Each axis of the sensor was oriented in the vertical position in both up and down directions, having a total of six configurations; the signal was acquired at each position of the sensor for 20 min. A tripod, with geodetic stage, was used to provide the support needed to make the plane supporting the sensor perfectly horizontal; a precision level was then used to adjust the orientation of the geodetic stage. It is important to avoid the presence of external agents like unexpected accelerations or temperature variations during calibration. The temperature, in particular, is the most difficult parameter to control because the sensor itself is subjected to heating during the functioning. To preserve the measurement under the influence of *run-to-run* component of bias, the sensor should not be turned off during calibration and the electric supply must be kept constant; to assure these conditions, the sensor was supplied using a stabilized power generator (0–20 V output).

**Figure 4.**
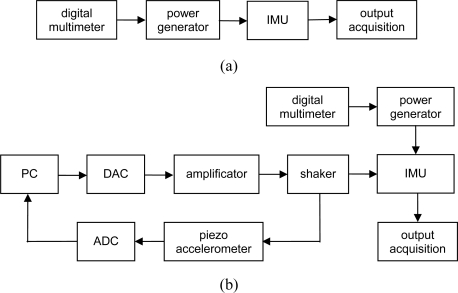
(a) Schematic of the experimental set-up for static calibration and AV estimation and (b) for the dynamic excitation step of the reliability test procedure (b).

**Figure 5.**
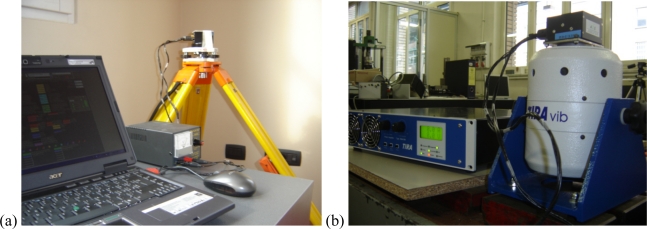
Experimental settings: (a) evaluation of static systematic and accidental errors by means of the six positions static test and the Allan variance calculation; (b) dynamic excitation step of the reliability test procedure.

The second step of the calibration is dedicated to the estimation of accidental errors through AV calculation. In this case, the sensor was simply subjected to a long-time acquisition (12 h) at a sampling rate of 100 Hz. The sensor was turned on and supplied for approximately 1 h before the starting of the acquisition, to avoid the influence of thermal drift on the output signal caused by the self heating of MEMS sensors. The accelerations provided by the three axes were stored and used to calculate the AV, as reported in the IEEE 952 normative [36]. The AV evolution in the time domain allows estimating systematic and accidental measurement errors at short, medium, and long time [38], as shown in Figure 1. A schematic of the experimental setup and an image of the experimental desk for the static calibration and AV calculation are presented in Figures 4a and 5a, respectively.

The second step of the reliability testing is represented by the dynamic excitation of the IMU. The vibration was imposed by using the electro-mechanical shaker, TIRA TV51120 (Figure 5b), and by following the spectrum profile described in the previous section. The temperature was assumed constant during the tests and equal to the ambient temperature. The IMU was connected to the shaker by a flange of steel; the input signal was represented by a sine sweep that follows the vibration spectrum. The software of control, LMS TestLab Tracked Sine Dwell, was used to drive the shaker, and the digital-to-analog converter (DAC) was used to convert the digital input signal to analog; the signal was then amplified and sent to the shaker. The level of vibration of the shaker was monitored by a closed-loop control. A piezoelectric accelerometer situated near the IMU provided feedback to the software of control by means of an analog-to-digital converter (ADC). The IMU was supplied at 10 V during the test and a digital multimeter was exploited to set the input voltage. The schematic of the test desk is presented in Figure 4b.

## 5. Reliability Test Results

### 5.1. Static Calibration

The values of bias and scale factors calculated before and after the dynamic excitation of the IMU are reported in Table 6. They represent the systematic errors affecting the measure resulting from the multiposition calibration procedure (six-position static test).

### 5.2. Allan Variance

**Figure 6.**
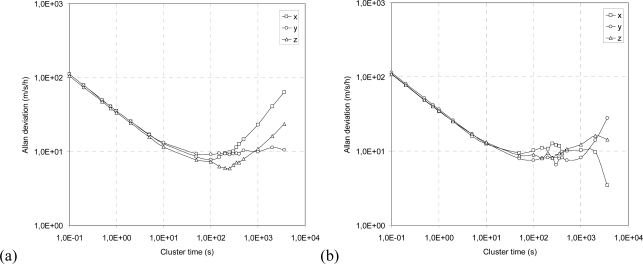
Allan deviation curves for acceleration signal calculated for each axis before (a) and after (b) the vibration test.

The Allan deviation calculated on each acceleration axis before and after the dynamic excitation is presented in double logarithmic scale in Figure 6a and 6b, respectively.

### 5.3. Signal Acquisition

To better understand the effects of dynamic excitation on signal characteristics, the acceleration was acquired and stored before and after the imposed vibration along each axis. The IMU was subjected to a dynamic displacement by means of the electro-mechanical shaker controlled by a feedback signal provided by a piezoelectric accelerometer. The actuation frequency used was limited by the sampling rate of the IMU (maximum of 100 Hz). The wave form can be traced properly by the IMU up to a frequency of 10 Hz, though the acceleration level can be measured at higher frequencies eventually. The signal of acceleration was acquired with three different approaches:

□ *Output stability over time*: Two values of vibration level were selected (0.3 and 0.6 g), each at the frequency values of 6 and 8 Hz; the output signal was stored for 30 s and its amplitude stability and wave form were checked.
□ *Low frequency characterization*: The vibration level was set to the value of 0.02 g at frequencies of 1.5 and 2 Hz; the signal was stored for 30 s and its amplitude stability and wave form were checked.
□ *Sine sweep*: Two values of vibration level were selected (0.3 and 0.6 g) and the frequency was first linearly increased from 5 to 10 Hz and then decreased from 10 to 5 Hz at the sweep rate of 0.5 Hz/s (the test was 10-s long).

**Figure 7.**
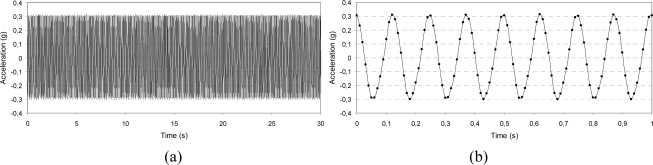
Signal stability before dynamic excitation: 0.3 g amplitude, 8 Hz frequency, x-axis; whole acquisition (a) and detail of the wave form (b).

**Figure 8.**
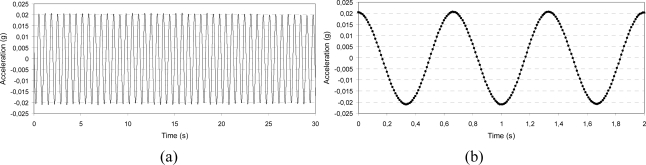
Low frequency characterization before dynamic excitation: 0.02 g amplitude, 1.5 Hz frequency, z-axis; whole acquisition (a) and detail of the wave form (b).

**Figure 9.**
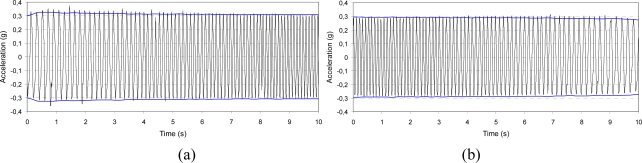
Sine sweep before dynamic excitation: 0.3 g amplitude, 0.5 Hz/s sweep ratio, y-axis. The horizontal lines correspond to the vibration level measured by the piezoelectric accelerometer used as feedback signal; increasing frequency 5–10 Hz (a) and decreasing frequency 10–5 Hz (b).

In Figures 7–9, some of the IMU output signals registered before the dynamic excitation are presented. For the sine-sweep test, the vibration level measured by the piezoelectric accelerometer situated on the feedback line was also traced.

**Figure 10.**
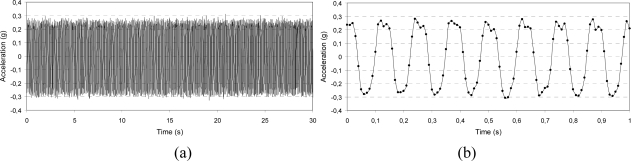
Signal stability after dynamic excitation: 0.3 g amplitude, 8 Hz frequency, x-axis; whole acquisition (a) and detail of the wave form (b).

**Figure 11.**
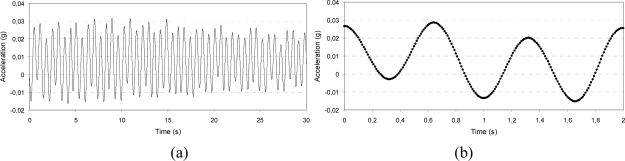
Low frequency characterization after dynamic excitation: 0.02 g amplitude, 1.5 Hz frequency, z-axis; whole acquisition (a) and detail of the wave form (b).

**Figure 12.**
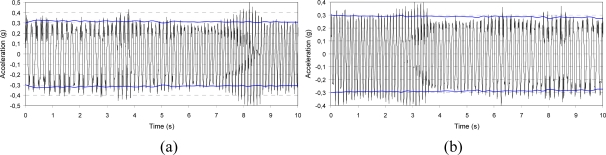
Sine sweep after dynamic excitation: 0.3 g amplitude, 0.5 Hz/s sweep ratio, y-axis. The horizontal lines correspond to the vibration level measured by the piezoelectric accelerometer used as feedback signal; (a) increasing (5–10 Hz) and (b) decreasing (10–5 Hz) frequency .

Figures 10–12 present the output acceleration signal after the dynamic excitation.

## 6. Discussion

Before calibration, the average acceleration of gravity, measured by the IMU among the three axes over a 15 min long acquisition period, was 9.848 m/s^2^. By applying the error model of equation (1), in which the measured biases and scale factors were inserted, the acceleration of gravity under the same conditions became 9.802 m/s^2^, *i.e.*, very close to its effective local value. This confirmed both the validity of the error model used and the correctness of the estimated biases and scale factors. An analysis of the results reported in Table 6 revealed how significantly the residual systematic parameters changed after the vibration test. Before the dynamic excitation, the standard deviations of the biases and scale factors were 0.252 and 0.025, respectively; after the dynamic excitation, the standard deviations became 2.955 and 0.087, respectively. This revealed that the measured sensing parameters were initially characterized by an excellent uniformity among the three axes, while this homogeneity was lost after the vibration test, especially for bias values.

Figure 6 shows the Allan deviation curves calculated on accelerations recorded by the sensor before and after the dynamic excitation. All the curves testify that after the imposed vibration, an increase in noise appears for high cluster times. With reference to the nomenclature shown in Figure 1, it is possible to observe an increase in the *bias instability* which is represented by the portion of the curve parallel to the abscissa axis. To quantify the increase in this portion of the curve, it is possible to identify a reference level σ = *c*·σ_min_ (where σ_min_ is the minimum value of the curve and *c* is a coefficient), and to consider the cluster time interval within the two intersections between this level and the curve. For instance, with $c=\sqrt{2}$, the cluster time interval is equal to 420 s before the vibration test and 945 s after the vibration test (average among the three axes), corresponding to an increase of 125% in the region of the curve identifying bias instability. This instability can be attributed to the increase in the uncertainty of the accidental error with respect to the medium-long period (cluster time greater than 100 s): this uncertainty involves an increase in the *run-to-run* bias component of the accelerometers. An additional indication of the performance variation after dynamic excitation is given by the shape of the Allan deviation curve at very long correlation times; these portions of the curves identify the errors called *rate random walk* and *drift rate ramp* (Figure 1), and their trends allow a qualitative estimation of increasing errors. From the Allan deviation curves, it appears that the vibration determines an increase the variability of these components. This indicates an additional component of noise whose nature is deterministic and is probably attributed to permanent modifications occurring inside the sensor.

The IMU performances tend to be less precise after the dynamic excitation: the output signal may no longer be accurate, the wave form may be irregular, and the vibration level may not be constant. An analysis of the acceleration signal has revealed an increase in the measurement errors and uncertainties in accordance with the indications provided by the Allan deviation diagrams.

Following the reliability procedure, the IMU was observed and analyzed: the integrity of the external package was controlled, the electric connections were verified and tested, and the MEMS sensing components were checked. A small deformation of the metallic frame was observed, probably produced by the effect of mechanical vibrations on its thin walls. This damage is potentially responsible for the variations in the sensing axes’ orthogonality and cross-coupling factors. With regard to electrical connections, several soldering failures were found, especially in larger and heavier components like capacitors. Sometimes, the connectivity of the joints was reduced, and in worst cases, the electrical component was completely detached. The larger components may probably have a resonance frequency comparable with the vibration spectrum excited during the dynamic test, determining very high accelerations acting on them. Combined with large masses, these accelerations produced the forces so relevant for causing component detachment. The decay of reliability caused by soldering weaknesses is a well-known issue, representing a key feature of devices functioning in the presence of mechanical vibrations and/or shocks [42]. With regard to the single MEMS components, no deteriorations or damages were observed; this confirms the high reliability of the small microsensors provided by the strength of the internal structures and the protective polymeric packaging.

The modifications observed inside the IMU confirm the response of the reliability test regarding the increase in error levels, and consequently, less accurate measurements. In particular, the bias and scale-factor variation detected by the calibration step of the procedure can be attributed to the less efficient signal processing, probably caused by the malfunction and detachment of some electrical components. The non-orthogonality factor variation is very probably owing to the metallic frame deformation and small misalignments between the frame and internal sensors. These misalignments depend on the small displacements of the anchored internal circuitry caused by the vibrations. All these effects are responsible for the increase in the accidental-errors uncertainty and the comparison of the deterministic errors. These errors can be detected by analyzing the Allan deviation curve at medium and long cluster times; they are associated with the sensor and appear as superimposed on the measurement.

## 7. Conclusions

In this study, a reliability procedure for IMU based on MEMS sensors for applications in aeronautics and vibrating environments has been presented; it is based on three steps and allows estimating the variation of important sensing parameters after the application of a dynamic excitation capable of reproducing the working conditions. Anomalous variations of the bias, scale factors, and non-orthogonality factors (especially their relative variation between the axes) are an indication of possible damaging processes might have occurred inside the sensor. The increase in the standard deviation among their values on *x*–*y*–*z* axes has been proved to be a valid indicator. The effects of internal malfunctioning are also observed to cause higher levels of inaccuracy and uncertainty of errors; these effects are detected by the presented procedure through the AV calculation. A variation of ±50% of the curve portion identifying the bias instability error must be considered physiological, because of the uncertainty of AV at medium-high cluster times. On the other hand, a more relevant increase in this region, like the one observed, is the consequence of permanent modifications (internal damages) causing additional noise components of the deterministic nature. The reliability procedure has been validated on a commercial IMU and the results provided by the test have been confirmed by a direct diagnosis of the sensor.

The main contributions of this study can be summarized as follows: (1) a reliability procedure for testing IMU based on MEMS operating in vibrating environments was presented; (2) a real IMU was studied according to the procedure described and the results provided by its analysis were presented; (3) the standard procedures were recalled as Allan variance calculation, six-position static calibration test, and aeronautic normative for vibration tests; (4) it was observed that the main reliability problems of the studied IMU are related to the electrical component connections, circuitry anchoring, and external frame flexibility; and (5) it was demonstrated that an IMU is less robust and reliable than the components used to build it, especially with regard to MEMS sensors.

## Figures and Tables

**Table 1.** Sensing performance requirements for IMU in three aerospace fields with increasing accuracy.

	Tactical grade	Navigation grade	Strategic grade
Error	>20 km/h	<1 km/h	<30 m/h
Gyro drift rate	1–10 deg/h	0.015 deg/h	0.0001 deg/h
Accelerometers bias	100–1000 μg	50–100 μg	1 μg
Costs of IMU	<10.000 $	10.000–70.000 $	>200.000 $

**Table 2.** Characteristics of vibration spectrum excitations for functional and endurance sections.

	Functional section		Endurance section	
	Level [PSD]	Sweep rate [Hz/s]	Level [PSD]	Sweep rate [Hz/s]
Broadband vibration	0.040	3.684	0.040	0.100
45–60 Hz band	0.100	0.067	0.100	0.067
300–400 Hz band	0.172	0.445	0.266	0.445
1,400–1,600 Hz band	0.172	0.890	0.266	0.890

**Table 3.** Test plan for the dynamic excitation.

	Functional section			Endurance section			Functional section		
Axis	x	y	z	x	y	z	x	y	z
Time	30 min	30 min	30 min	5 h	5 h	5 h	30 min	30 min	30 min

**Table 4.** Main characteristics of the AXIS-AIS402 inertial measurement unit.

Dimensions	70 × 60 × 40 mm
Fixing flange dimensions	78 × 66 mm
Weight	230 g
Supply voltage	9–30 V
Current	175 mA at 9 V

**Table 5.** Sensing specifications of the AXIS-AIS402 inertial measurement unit.

	Measurement field	Static precision	Dynamic precision	Resolution	Noise	Bandwidth
Angular velocity	±150°/s	±0.5°/s	-	0.07°/s	0.07°/s	5 Hz
Acceleration	±2/±15 g	(±20/±100)·10^−3^ g	-	(1/9)·10^−3^ g	(1/12)·10^−3^ g	5 Hz
Roll	±180°	±1.5°	±4°	0.025°	0.15°	5 Hz
Pitch	±90°	±1°	±4°	0.012°	0.10°	5Hz
Yaw/Heading	0–360°	±3°	±4°	0.025°	0.5°	-
Velocity	±1,200 km/h	±0.2 m/s	-	0.05 m/s	0.2 m/s	-
Altitude	−0.6–8 km	5 m	-	1 m	1 m	-
Positioning	-	3 m	-	0.01 m	1 m	-

**Table 6.** Acceleration biases (bi) and scale factors (Si) for each axis of the IMU before and after the vibration test.

	Before	After
b_x_	−4.9 mg	−6.8 mg
b_y_	−4.4 mg	−8.3 mg
b_z_	−4.7 mg	−2.6 mg
S_x_	−0.02 %	−0.14 %
S_y_	−0.07 %	0.01 %
S_z_	−0.05%	−0.14%

## List of symbols

*a*: acceleration
*b_a_*: acceleration bias
*b_ω_*: angular velocity bias
*K*: rate random walk coefficient
*l_a_*: measured acceleration
*l_ω_*: measured angular velocity
*N*: number of samples
*R*: drift rate ramp coefficient
*S*: power spectral density
*S_a_*: acceleration scale factor (gain)
*S_ω_*: angular velocity scale factor (gain)
*T*: non-orthogonality factor
*dγ*: acceleration of gravity variation
ε*_a_*: acceleration noise
*ε_ω_*: angular velocity noise
γ: acceleration of gravity
*σ*: Allan deviation
τ: cluster time
*τ_0_*: sampling period
Ω: output signal
*ω*: angular velocity
*ω_e_*: earth angular velocity
